# A neural tracking and motor control approach to improve rehabilitation of upper limb movements

**DOI:** 10.1186/1743-0003-5-5

**Published:** 2008-02-05

**Authors:** Michela Goffredo, Ivan Bernabucci, Maurizio Schmid, Silvia Conforto

**Affiliations:** 1 Dipartimento di Elettronica Applicata, Università degli Studi "Roma TRE", Roma, Italy

## Abstract

**Background:**

Restoration of upper limb movements in subjects recovering from stroke is an essential keystone in rehabilitative practices. Rehabilitation of arm movements, in fact, is usually a far more difficult one as compared to that of lower extremities. For these reasons, researchers are developing new methods and technologies so that the rehabilitative process could be more accurate, rapid and easily accepted by the patient. This paper introduces the proof of concept for a new non-invasive FES-assisted rehabilitation system for the upper limb, called smartFES (sFES), where the electrical stimulation is controlled by a biologically inspired neural inverse dynamics model, fed by the kinematic information associated with the execution of a planar goal-oriented movement. More specifically, this work details two steps of the proposed system: an *ad hoc *markerless motion analysis algorithm for the estimation of kinematics, and a neural controller that drives a synthetic arm. The vision of the entire system is to acquire kinematics from the analysis of video sequences during planar arm movements and to use it together with a neural inverse dynamics model able to provide the patient with the electrical stimulation patterns needed to perform the movement with the assisted limb.

**Methods:**

The markerless motion tracking system aims at localizing and monitoring the arm movement by tracking its silhouette. It uses a specifically designed motion estimation method, that we named Neural Snakes, which predicts the arm contour deformation as a first step for a silhouette extraction algorithm. The starting and ending points of the arm movement feed an Artificial Neural Controller, enclosing the muscular Hill's model, which solves the inverse dynamics to obtain the FES patterns needed to move a simulated arm from the starting point to the desired point. Both position error with respect to the requested arm trajectory and comparison between curvature factors have been calculated in order to determine the accuracy of the system.

**Results:**

The proposed method has been tested on real data acquired during the execution of planar goal-oriented arm movements. Main results concern the capability of the system to accurately recreate the movement task by providing a synthetic arm model with the stimulation patterns estimated by the inverse dynamics model. In the simulation of movements with a length of ± 20 cm, the model has shown an unbiased angular error, and a mean (absolute) position error of about 1.5 cm, thus confirming the ability of the system to reliably drive the model to the desired targets. Moreover, the curvature factors of the factual human movements and of the reconstructed ones are similar, thus encouraging future developments of the system in terms of reproducibility of the desired movements.

**Conclusion:**

A novel FES-assisted rehabilitation system for the upper limb is presented and two parts of it have been designed and tested. The system includes a markerless motion estimation algorithm, and a biologically inspired neural controller that drives a biomechanical arm model and provides the stimulation patterns that, in a future development, could be used to drive a smart Functional Electrical Stimulation system (sFES). The system is envisioned to help in the rehabilitation of post stroke hemiparetic patients, by assisting the movement of the paretic upper limb, once trained with a set of movements performed by the therapist or in virtual reality. Future work will include the application and testing of the stimulation patterns in real conditions.

## Background

Rehabilitative practice in stroke survivors has strengthened its empirical foundation on the basis of the recent advances in neuroscience methods, which led to deeper understanding of motor control and learning mechanisms, also on the basis of the recent discoveries regarding cells injury and regeneration [1]. In particular, long-term potentiation (i.e. where synapses are able to encode new information to represent a movement skill) is considered to have a key-role for the restoration of impaired functions. A critical element for the success of these mechanisms resides in the repetition of similar inputs for the motor cortex: these inputs, in fact, act as a biological teacher for the neuronal acquisition of novel skills. This process could be easily implemented through experience and training, which induce physiological and morphological plasticity, by strengthening synaptic connections between neurons encoding common functions [2]. Thus, the key concept behind the neurological rehabilitation is the repetition of movements in a learning-by-examples paradigm: by repeating movements, in either passive or assisted way, the brain is exposed to reinforcement and the neurons strengthen their connections.

To accomplish this purpose, the restoration of motor functions in people recovering from cerebrovascular diseases is typically achieved by means of adaptive equipments and environmental modifications [3,4]. Significant improvement is being made in understanding the cellular and molecular events of cell injury and regeneration, and the paradigm of the massive repetition of movements to strengthen functional outcome is a necessity thus forcing new clinical treatments to exploit these new discoveries [5-10].

Functional Electrical Stimulation (FES) is one of the most used technologies for restoring the functions of patients affected by neurological pathologies. By electrically activating the muscular system, FES is increasingly recognised as therapy and treatment for subjects impaired by stroke, multiple sclerosis and cerebral palsy [11,12]. The electrical stimulation has overcome the simple functional limb substitution [13], and has been proved as a successful therapy tool both in lower [14] and in upper limb movements [15]. These encouraging results have recently brought to the development of FES-assisted rehabilitation programs for hemiplegic patients [16], thus disclosing the idea of functional electrical therapy, FET [17]. Currently, FET systems make use of residual motor functions [18] or EMG recordings from muscular activity [19]. Recent technologies include non-invasive stimulators, like the handmaster [20] and the bionic glove [21], but the presence of external devices does not appear desirable for patients with neurological injuries. Novel and more sophisticated technologies, able to convey FES-based rehabilitation programs in an automatic and non-invasive way, are thus needed.

Following this objective, a new non-invasive FES-assisted rehabilitation system for the upper limb is here presented: the electrical stimulation is controlled by a biologically inspired neural inverse dynamics model, fed by the kinematics associated with the execution of a planar goal-oriented movement. The system, called smartFES (sFES), exploits the recent neurological discoveries on the effect of the repetition of rehabilitation exercises for the recovery of motor functions in stroke survivors.

**Figure 1:**
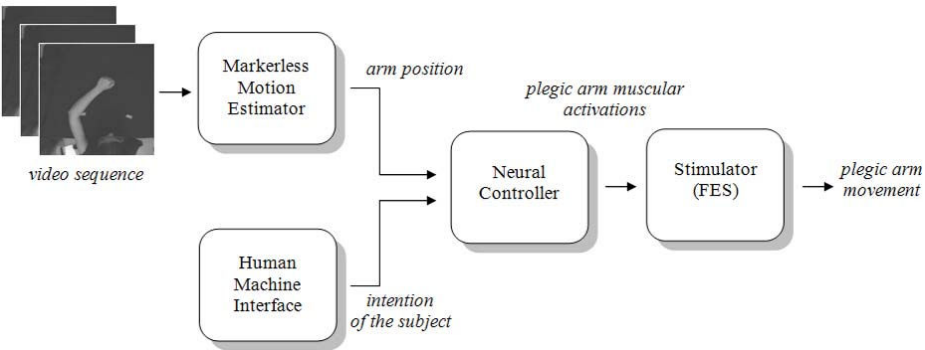
Block diagram of the proposed system.

Figure 1 shows the flow diagram of sFES, which is composed by four main blocks. The first block uses a markerless analysis to track the position of the healthy arm. This is being accomplished without using any kind of sensor or marker applied to the patient. In the second block a human machine interface (HMI, not discussed here), based on subject gaze interpretation, gives information regarding the intention of the subject (that is, where the arm has to go to). A neural controller then uses this information, regarding "where the arm is" and "where it is going to", to generate the specific outputs. These outputs are the muscular forces which are necessary for the execution of the specific movement via the FES block that provides the corresponding electrical stimulation.

As a proof of concept, in the current paper only the markerless silhouette tracking algorithm and the neural controller for the execution of point to point planar movements of the upper limb will be presented. In fact, these steps are crucial in designing a system which could help patients in recovering movements through FES, because they estimate the movement and solve the inverse problem in terms of the pattern of stimulation needed for accomplishing the desired movement. For the HMI different approaches are possible, while the implementation of the FES stimulator is the next step in our research program.

The use of a markerless motion estimation method for controlling a FES-based rehabilitation exercise is a novelty in the research area. There is a rich literature on various human motion analysis techniques which estimate limb poses from video sequences for different purposes, like video surveillance [22], human-computer interaction [23], gesture recognition [24] and biomechanical applications [25]. The approaches can be grouped in model-based and model-free methods (see [26] for a review). The first group uses human body models in order to estimate the limb poses from the video sequences. These approaches generally need more than one video camera capturing the movement of the entire body and present high computational costs. On the other hand, model-free methods rely on the motion estimation of single pixels belonging to the body limbs [27], or on the extraction of the body silhouette [28]. The first approach is basically sensitive to noise and light changes, and needs an initialization phase for the selection of the pixels of interest. Conversely, the silhouette-based motion estimation appears a good compromise between computation time, automatism and robustness. Edge detectors [29] can be extracted robustly and at low cost, but they are unsuitable to deal with cluttered backgrounds or textured clothing. Therefore, silhouettes are usually located with contour or shape approaches that are more accurate than edge detecting techniques in tracking non-deformable objects [30,31]. A recent optimization of the Snake algorithm [32], which allows to extract the silhouette of deformable objects, like human body limbs, had been proposed by the authors of this paper [33]. The method, called Neural Snake, appears to be a sound choice for the sFES system where a trade-off between computation time and accuracy is needed.

The second block of the sFES system is a biologically inspired controller of the stimulation waveforms for the arm. Even though some pioneering work has been found in literature [34], a neural FES controller has not yet been deeply investigated. For this purpose, Artificial Neural Networks (ANN), which have been firstly hypothesized as biologically reasonable controllers [35], are proven to be an efficient tool for the resolution of the inverse kinematics [36]. The aim of the ANN is therefore to replace a controller activated step-by-step by the patient (for instance, with the contraction of residual muscles) with a high level motor controller driven by the action to be implemented (i.e. move the arm from position A to B, reach an object and so on) [37]. For this purpose, after receiving the information regarding the desired movement, the stand alone neural controller drives the stimulator block to make the assisted arm move in the requested way. Therefore, the rehabilitation exercise will be composed of movements shown by a "healthy teaching arm" and reproduced by means of the neural driven sFES.

## Methods

### The markerless motion estimation method

The first step of the proposed sFES system is the markerless motion estimation of the healthy arm pose. For this purpose, silhouette approaches, like Active Contour Models (Snakes), offer a partial solution because they imply that the shape has to be preserved during the movement. However, in human movement analysis it is often needed to track silhouettes whose shape is largely changing from frame to frame, and dealing with this issue is increasingly more demanding in presence of low acquisition frame rates with respect to the velocity in the execution of movement. Therefore, in order to apply the Snake algorithm in the dynamic context, the present study introduces a new approach, called Neural Snakes (NS).

The algorithm is based on the design of a specific ANN (ANN1 in the following) which works in a two-steps basis: firstly, it predicts the deformation of the contour shape on a frame by frame basis, then this coarse estimation of the silhouette position is refined by means of the Snake algorithm described in [32]. In this way, the NS algorithm is able to track the changes in the silhouette far better than the simple Snake algorithm.

**Figure 2:**
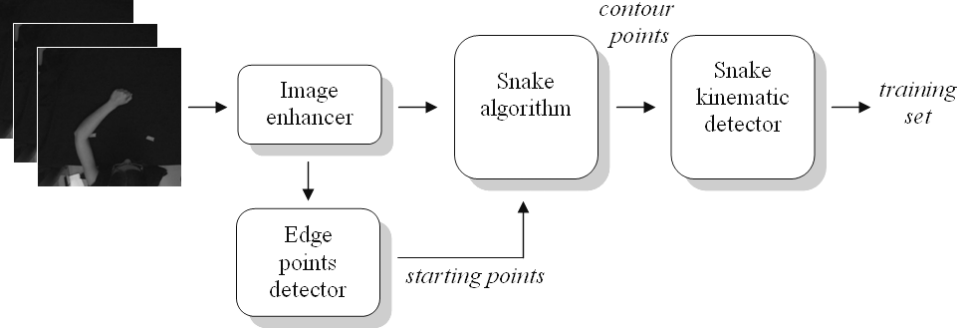
Graphical representation of the proposed algorithm to obtain the training set of the Neural Snake system.

In the following, the different phases composing the markerless motion estimator are described, with reference to Figure 2.

**Figure 3:**
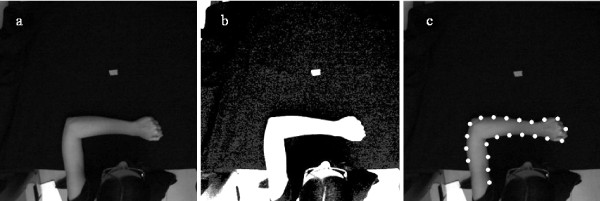
66th frame of one of the video sequences used for training ANN1. a) Original frame. b) Frame after the application of the image enhancer. c) Points obtained after the sub-sampled edge detector.

The video sequences, used in the training phase, have been captured in a controlled environment where upper limb movements are executed under constant and bright lights. The image contrast has been increased by using specific libraries from a video capture/processing utility [38] and successively a sharpening filter has been applied (Figure 3). Then, after filtering through a 5-by-5 median filter, the arm silhouette is extracted as reported in Canny [39] and uniformly sub-sampled (for frames shown in Figure 2, the number of points is 22).

The edge points are then used as contour points (CP) for the Snake algorithm where their matching to the image contour is achieved by minimizing a cost function, defined "energy". As explained in [32], the contour is a controlled discrete spline function that can be parametrically represented by a sequence of samples **v**(*s*):

**v**(*s*) = (*x*(*s*), *y*(*s*))

The energy expression, in case of *N *contour points CP(*i*) (*i *= 1,..., *N*), where the samples **v**(s) are evaluated at *s *= *s*_*i*_, is the following:

$$E_{tot}=E_{int}+E_{ext}=\sum_{i=1}^{N}E_{CP\left(i\right)}$$

where the internal energy *E*_*int *_can be written as a function that includes the inter-points distance and the contour curvature

$$E_{int}=\frac{\alpha {\left|\frac{dv}{ds}\right|}^{2}+\beta {\left|\frac{dv^{2}}{ds^{2}}\right|}^{2}}{2}$$

and where *α *and *β *are respectively the measure of elasticity and stiffness of the Snake. The first derivative term makes the Snake act like a membrane, where the constant *α *controls the tension along the contour. On the other hand, the constant *β *and the second order term drives the rigidity of the curve (if *β *is zero, the contour is discontinuous in its tangent, i.e. it may develop a corner at that point).

The external energy of the Snake, *E*_*ext*_, is derived from the image data to make the Snake be attracted to lines, edges and terminations:

*E*_*ext *_= *E*_*line *_+ *E*_*edge *_+ *E*_*term*_

where

$$\begin{array}{l} E_{line}\propto f(x,y)\\ E_{edge}\propto {\left|\nabla f(x,y)\right|}^{2}\\ E_{term}\propto \frac{\partial \theta (x,y)}{\partial n_{r}} \end{array}$$

and *f*(*x*, *y*) is the image intensity, *θ*(*x*, *y*) is the gradient direction along the contour and *n*_*r *_is an unit vector perpendicular to the gradient direction.

The application of the described energy minimizing procedure on the *N *contour points extracted from the controlled video sequences generates the training set of the ANN1. The resulting horizontal and vertical positions of the contour points, together with their velocities and accelerations, are used to estimate the dynamics of shape.

**Figure 4:**
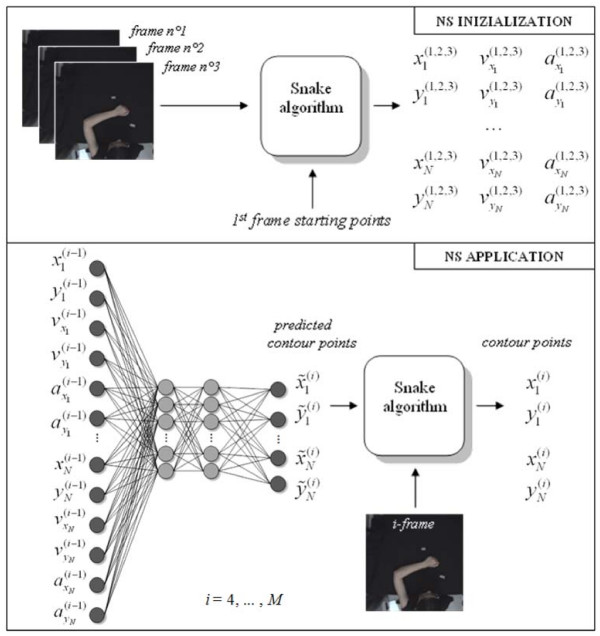
Graphical representation of the proposed algorithm for the upper arm silhouette tracking.

ANN1 is a Multi-Layer Perceptron composed of 2 hidden layers that are composed of 15 neurons each. This configuration has been chosen after a trial-and-error optimisation with respect to complexity, accuracy and real-time implementation. The network is fed by the horizontal and vertical components of position $\left(x_{n}^{\left(i-1\right)},y_{n}^{\left(i-1\right)}\right)$, velocity $\left(v_{xn}^{\left(i-1\right)},v_{yn}^{\left(i-1\right)}\right)$ and acceleration $\left(a_{xn}^{\left(i-1\right)},a_{yn}^{\left(i-1\right)}\right)$ of all the contour points *n *(*n *= 1,..., N) in the current frame (*i*-1) (which means that the number of the input neurons is *N *× 6). The (*N *× 2) outputs are given by the horizontal and vertical components of the position of the contour points in the subsequent frame *i*. For the training phase, a Resilient Back Propagation algorithm has been chosen. At the end of the training of ANN1 (2000 epochs were necessary for convergence), the network is used as a shape contour predictor for the arm motion estimation in an uncontrolled environment. Both the ANN1 training procedure and its application in the NS method are shown in Figure 4.

Therefore, in an uncontrolled video sequence, the arm movement is firstly predicted by the trained ANN1 and then corrected with a fine estimation by using the Snake algorithm. For each frame *i*, the output of the predictor (the *N *predicted contour points) and the *i*-th frame of the video sequence are processed by the Snake algorithm in order to minimise eq. (2). The result is the silhouette pose estimation over time. Moreover, the CP positions obtained with the NS approach allow the estimation of elements characterizing the kinematics of the gesture, such as the position of the wrist as the end point, or the shoulder joint behaviour.

The NS had been previously tested on synthetic video sequences with different values of signal-to-noise ratio (SNR) [33] in order to evaluate its accuracy: the results showed a RMSE lower than 1 cm and almost independent on the SNR. Moreover, comparative tests on real video sequences had shown the improvement of NS in tracking deformable shapes as compared to the Snake algorithm.

Therefore, the proposed markerless silhouette tracking algorithm can be confidently used for the estimation of the arm position, which drives, together with a HMI, the neural motor controller.

### The neural controller of the upper limb model

**Figure 5:**
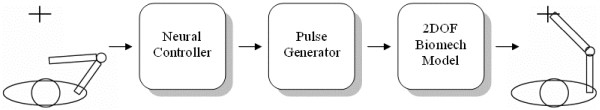
Graphical representation of the proposed method for the neural controller of the upper limb model.

The trajectory parameters extracted by the NS algorithm are used to drive a neural controller which activates a biomechanical model of a simulated human arm and controls the FES. To this purpose, and according to [36], a second ANN (ANN2 in the following) implements the neural controller which solves the inverse dynamic problem. Once the movement intended by the subject is known (it can be simply specified in terms of starting and ending coordinates of the movement and provided by the HMI), the controller generates the neural activations that will make the artificial muscles produce the forces necessary to drive the arm model. The outputs of ANN2 are used by the so-called Pulse Generator block, which builds the waveforms needed to generate the muscular activations. The scheme of the whole controller is shown in Figure 5.

The arm model is composed of two joints (two degrees of freedom) and four muscle-like actuators (agonist and antagonist pair for both shoulder and elbow joint), which execute the planar movement on the basis of the muscular activations. The skeletal structure of the simulated biomechanical arm consists of two segments, with lengths L1 and L2, which represent the forearm and the upper arm respectively, connected through two rotoidal joints. The planar joints that connect the two segments can assume angular values in the range [*0*, *π*]. These values are in correspondence with the Cartesian coordinates of the free end in the working plane by means of well known direct kinematic transformation. The muscular structure is simulated by means of four Hill's type muscle-like actuators, and establishes the dynamic relationship between the position of the arm and the torques acting on each joint [40]. Body segment anthropometrics and inertias of both upper arm and forearm are obtained from the scientific literature, taking into account the specific body height and weight [41].

**Figure 6:**
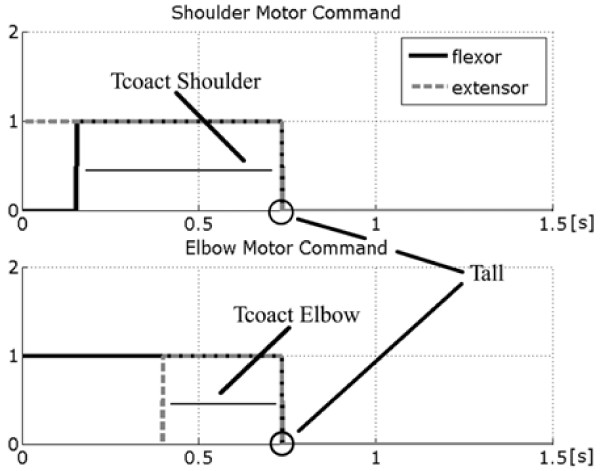
Neural activations of both the shoulder and the elbow muscle pair. Tall is the total time of neural activations, the same for the two joints; the two Tcoact values represent the interval of co-activation of flexor and extensor muscle. The value of 1.5 s is the total observation time.

ANN2 has been designed by using a Multi-Layer Perceptron with two hidden layers, fed by four inputs, representing the coordinates of the starting and the ending points of the movement to be generated. The hidden layers are composed by 20 neurons each, while the output layer gives three values representing respectively: the time of co-contraction of the muscular pairs of both the shoulder (T_*coact shoulder*_) and the elbow joint (T_*coact elbow*_), together with the duration of the overall neural activations (*T*_*all*_). These parameters are provided to the Pulse Generator block, which transforms them in a train of efferent nervous spikes necessary to drive the movement. Figure 6 depicts the profile of these neural activations having rectangular shapes, and shows the duration of the entire voluntary task ranging in the interval 300 ms – 1 s.

The network has been trained by a Resilient Back Propagation algorithm. Around 200000 epochs are necessary to train ANN2. Details on the implementation of the neural controller can be found in [36].

Two steps of the non-invasive FES-assisted rehabilitation system for the upper limb have been presented. In synthesis, once acquired a video sequence of an healthy arm movement, the neural controller makes it possible to extract the muscular activations that are necessary to make the synthetic arm execute the presented task.

## Experimental tests

**Figure 7:**
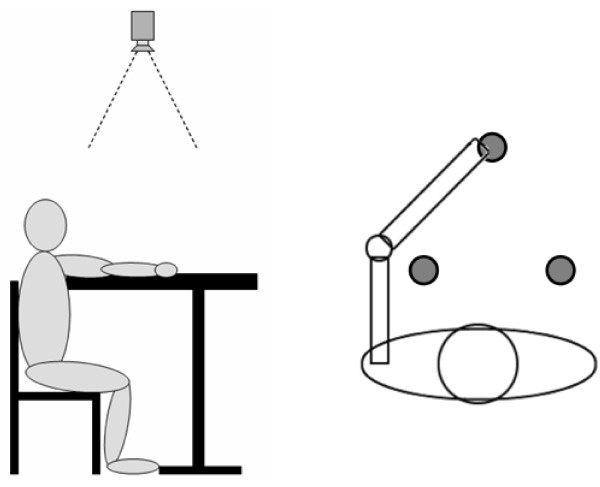
Experimental setup for the markerless estimation algorithm.

The experimental tests have been done by recruiting 2 healthy subjects. During tests, the subject sits on a chair in front of a desk whose height is the same of the subject's armpit. By reaching different points on the desk surface, it is thus possible to approximate the overall upper limb kinematics through its projection on to the desk plane. Three target points are set on the table surface and a digital video camera (Silicon Imaging MegaCameras SI-3300RGB) records the movements from an upper view with spatial resolution 1024 × 1020 pixels. The experimental protocol consists of a series of 3 fast reaching movements (a triangle) executed with the "healthy teaching arm" towards three different targets, considering the centre of the closed hand as the end-effector. In the experimental tests, the left upper limb has been considered as the "healthy teaching arm". Figure 7 shows the experimental setup.

The video sequence used for training the Snake predictor ANN1 has been acquired at 60 fps and the arm movements have been executed slowly.

After the ANN1 training phase, two video sequences of natural arm movements have been acquired at 30 fps (frame rate commonly used in commercial low costs digital cameras), the proposed NS method has been applied and the close hand and shoulder positions have been estimated over time.

Experimental trials have been performed for assessing the capability of the neural controller to make the synthetic arm execute movements corresponding to those determined by the markerless algorithm. In order to evaluate the performance of the two system blocks, a number of parameters have been extracted from the trajectories of the different movements.

The Cartesian coordinates of the three targets reached by the subject's arm have been expressed in a reference system centred in the shoulder, and the obtained values have been fed to the neural controller. Both the starting and the ending points of the three trajectories have been estimated via the NS algorithm. For each pair of points, ANN2 has been run to generate the neural excitations that enable the biomechanical arm model to execute a movement similar to the video-acquired one.

Indicating with *P*_*j *_= (*p*_*xj*_, *p*_*yj*_) the horizontal and vertical coordinates of the target point *j *(*j *= 1, 2, 3) and with ***T***_***j ***_= (***t***_***xj***_, ***t***_***yj***_) the trajectory executed leading to the target point *j*, two measures have been considered as indexes of accuracy of the NS algorithm and the neural controller.

Firstly, the mean absolute value of the position errors (*PE*_*j*_) between the true and the estimated (by the NS) *j*-th target position has been considered:

$$PE_{j}=\sqrt{{\left(p_{xj}^{e}-p_{xj}^{t}\right)}^{2}+{\left(p_{yj}^{e}-p_{yj}^{t}\right)}^{2}}$$

where the estimated target position is indicated with the superscript *e *and the true one with the superscript *t*.

Subsequently, the difference between the estimated (by the NS) and the reconstructed (by the neural controller) trajectory have been evaluated by extracting the error trend, calculated in the following way for each trajectory ***T***_***j***_:

$$E_{j}=\sqrt{{\left(t_{xj}^{e}-t_{xj}^{r}\right)}^{2}+{\left(t_{yj}^{e}-t_{yj}^{r}\right)}^{2}}$$

where the estimated trajectory is indicated with the superscript *e *and the reconstructed one with the superscript *r*.

Furthermore, the curvature of the reconstructed movements has been chosen as a measure for evaluating the system performance. In literature, it is reported that ballistic natural movements are typically smooth and with a limited curvature [42-44]. According to the definition of curvature reported by [42], we used the ratio between the trajectory length and the Euclidean distance between the starting point and the arrival point:

$$C_{j}=\frac{L\left(T_{j}\right)}{\sqrt{{\left(p_{xj}-p_{xj+1}\right)}^{2}+{\left(p_{yj}-p_{yj+1}\right)}^{2}}}$$

where the numerator is the length of the *j*-th trajectory (composed of *H *points)

$$L\left(T_{j}\right)=\sum_{h=1}^{H-1}\sqrt{{\left(dt_{xj}^{h}\right)}^{2}+{\left(dt_{xj}^{h}\right)}^{2}}$$

and the denominator is the distance between the starting and arrival points of the *j*-th trajectory.

## Results

**Figure 8:**
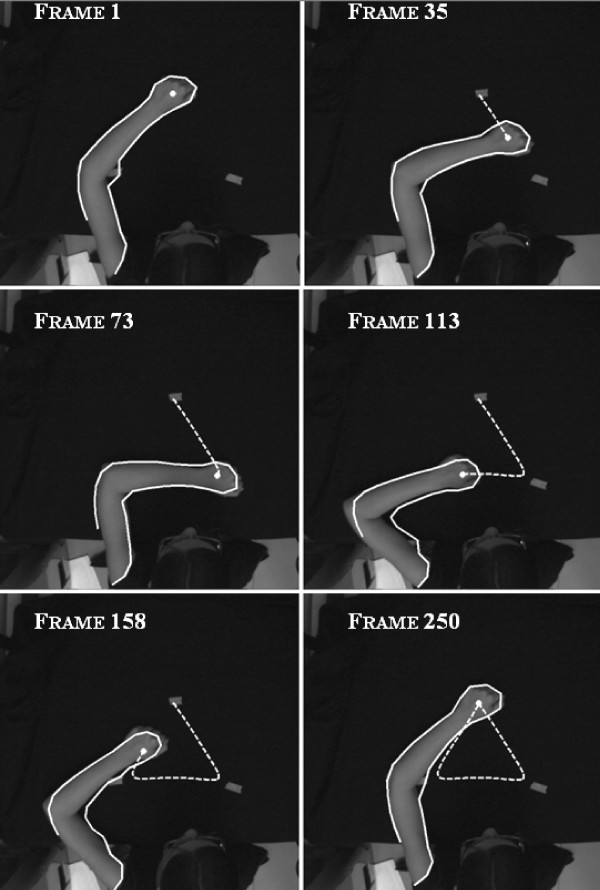
Upper limb silhouette estimation by means of the Neural Snake (solid line) and close hand estimated trajectory (dot line) on some relevant frames of the video sequence.

Figure 8 shows the results of the proposed silhouette detector and the obtained trajectories on selected frames of the video sequence: the NS algorithm successfully extracts the arm movement.

**Figure 9:**
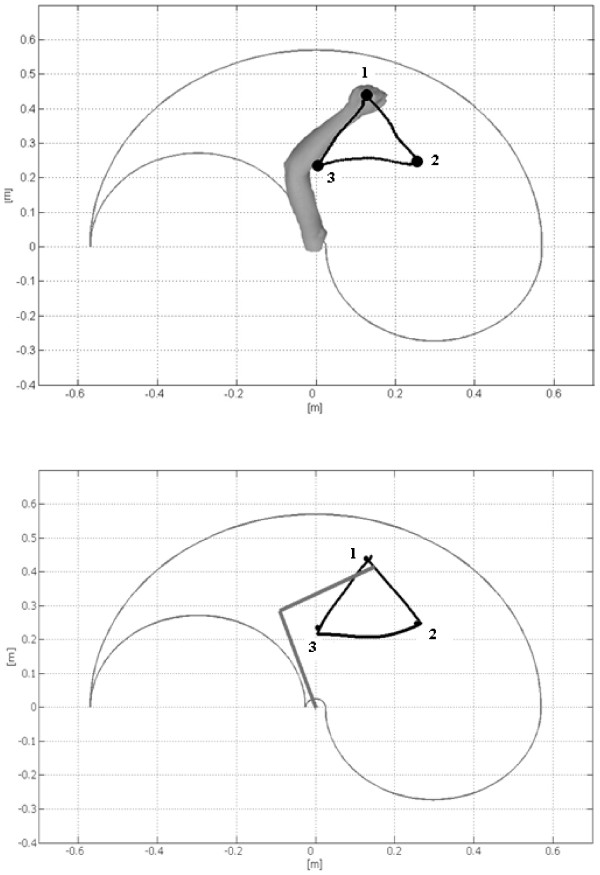
Close hand estimated trajectory (up) and output of the Neural Controller (down) that provides the reconstructed arm trajectory.

The end-effector positions, estimated by NS, are then provided to the neural controller, see Figure 9, where the two triangular trajectories are compared. From these results it emerges that any image containing the arm movement can reliably feed the neural controller to drive the biomechanical model.

The *PE*_*i*_, as defined in (6), is presented in table 1: the obtained figures, with a mean value of 1.5 cm, have the same order of magnitude of the ones obtained with synthetic video sequences in [33]. Therefore, the results obtained with the proposed markerless arm motion estimation method are encouraging.

**Table 1:** 

**Via-points of the overall trajectory**	***PE*_*i *_(cm)**
*P*_1_	1.12
*P*_2_	1.96
*P*_3_	1.34

**Figure 10:**
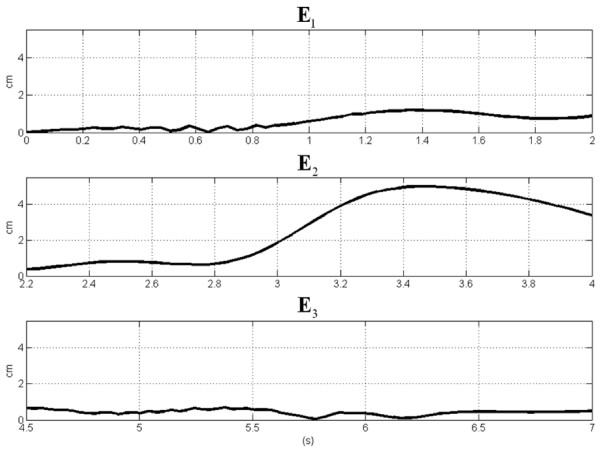
Error trends for each movement of the triangular trajectory.

The error **E**_*j *_between the estimated and the reconstructed *j*-th trajectories is shown in Figure 10. The error is lower than 1.5 cm for the 1^st ^and the 3^rd ^movement. The increased value obtained for the middle movement could be linked with the inherent increased variability due to the non zero velocity in correspondence to that point.

Finally table 2 shows the comparison between the estimated values and the reconstructed ones in terms of curvature, following the (8).

**Table 2:** 

**Single segments of the overall trajectory**	**Curvature of the estimated movement**	**Curvature of the reconstructed movement**
**T**_1_	1.0061	1.0005
**T**_2_	1.0305	1.0241
**T**_3_	1.0583	1.0051

The mean curvature is 1.03 for the movements estimated by NS and 1.06 for the ones reconstructed by the neural controller. These values are in accordance to the results reported in [42]. Therefore, the obtained movements show a good agreement, not only for the final points but also for the trajectory.

## Conclusion

The proof of concept of a new non-invasive FES-assisted rehabilitation system for the upper limb has been presented. In the system, called smart FES (sFES), the electrical stimulation, necessary to assist a goal-oriented planar movement of one upper limb, is controlled by a biologically inspired neural controller, a HMI and a healthy arm motion detector. Four main blocks compose the overall system. The first one is dedicated to the markerless analysis of the healthy arm during planar movements from which information regarding the trajectory and the current position of the "healthy teaching arm" is obtained. In the second block a HMI based on gaze tracking and interpretation (not described here) will give information regarding the intention of the subject (which position the arm is going to). Then, the neural controller uses the outputs of the first and the second blocks for generating the specific outputs, which are the stimulations necessary to make the FES-assisted arm execute the movement.

In this paper, the markerless motion estimation method and the neural controller have been presented. The approach has been tested on real data and comparative results between the real, the estimated and the reconstructed target positions have been reported. Experimental results shows mean errors lower that 2 cm and are particularly encouraging for the future development of the system. Moreover, from the comparison of the arm trajectories estimated by the markerless algorithm and the ones reconstructed by the neural controller it emerged that curvature indexes are comparable and in accordance with the values found in literature.

In the future, the other two blocks of sFES will be designed. In particular, a HMI system, based on gaze identification, will process the subject motion intention in order to define the arm trajectory. Moreover, the neural controller outputs will be used to generate the electrical stimuli of the FES system that will make an assisted arm perform rehabilitation exercises.

## Competing interests

The author(s) declare that they have no competing interests.

## Authors' contributions

Michela Goffredo was responsible of the markerless part of the research project and of writing the paper. Ivan Bernabucci performed the motor control analysis and assisted with the study. Maurizio Schmid made conceptual contributions and supervised all aspects of its implementation. Silvia Conforto was the project leader and was responsible for the overall supervision. All authors read and approved the final manuscript.
